# Retraction: Regulation of BAD by cAMP-dependent protein kinase is mediated via phosphorylation of a novel site, Ser155

**DOI:** 10.1042/BJ3490547_RET

**Published:** 2025-01-30

**Keywords:** apoptosis, Bcl-2, cAMP, MAP kinase, protein kinase B

This article “Regulation of BAD by cAMP-dependent protein kinase is mediated via phosphorylation of a novel site, Ser155” (DOI: 10.1042/bj3490547) is being retracted from the Biochemical Journal at the request of the authors following receipt of a notification from a reader, alerting the Editorial Office to similarities in Western blot images between several figures, namely:

- Figure 3B, areas of similarity within the figure
- Figure 4A BAD (forskolin and IBMX treatment) and Figure 9B BAD (TPA, forskolin and IBMX treatment)
- Figure 4A Ser155 (forskolin and IBMX treatment) and Figure 9B Ser155 (TPA, forskolin and IBMX treatment)
- Figure 4A Ser112 (forskolin and IBMX treatment) and Figure 7 Ser112 (IGF and wortmannin treatment)
- Figure 4B Ser136 (forskolin, H89 and Ro318220 treatment) and Figure 6B BAD (TPA, PD98059, U0126, Ro318220 treatment)
- Figure 4C BAD (forskolin, PD98059, rapamycin and wortmannin treatment) and Figure 6B BAD (TPA, wortmannin, rapamycin and Ro318220 treatment)
- Figure 5A Ser155 (EGF treatment) and Figure 7 Ser155 (IGF and wortmannin treatment)
- Figure 5B BAD (TPA treatment) and Figure 7 BAD (IGF and wortmannin treatment)

The corresponding author and the research integrity group in the School of Life Sciences at the University of Dundee independently analysed the issues raised and agreed with the concerns and, with the agreement of the co-authors, proposed to the journal that the paper should be retracted.

The authors remain confident in the results presented in Figures 1 and 2 and Table 1, and the key finding that cAMP-dependent protein kinase (PKA) phosphorylates mouse BAD at serine 155 *in vitro* has been confirmed independently by others [Virdee et al, 2000 (DOI: 10.1016 /S0960-9822(00)00702–8) and Zhou et al, 2000 (DOI: 10.1074/jbc.M002526200)].

The other key finding noted in this article that forskolin, acting via cAMP and PKA, stimulates phosphorylation of BAD at serine 155 in cells has been confirmed by several laboratories [Tan et al, 2000 (DOI: 10.1074/jbc.M004199200), Zhou et al, 2000 (10.1074/jbc.M002526200) and Datta et al, 2000 (DOI: 10.1016 /S1097-2765(05)00012–2)].

The authors sincerely apologise for any inconvenience caused. The Editor-in-Chief and Editorial Board agree with the decision to retract the article.

